# First person – So-Hyun Lee

**DOI:** 10.1242/dmm.052504

**Published:** 2025-06-20

## Abstract

First Person is a series of interviews with the first authors of a selection of papers published in Disease Models & Mechanisms, helping researchers promote themselves alongside their papers. So-Hyun Lee is first author on ‘ Mutant zebrafish lacking *slc25a22a* show spontaneous seizures and respond to the anti-seizure medication valproic acid’, published in DMM. So-Hyun is a research professor in the lab of Seok-Yong Choi at Chonnam National University Medical School, Hwasun, Republic of Korea, investigating the molecular pathogenesis of epilepsy using a zebrafish model.


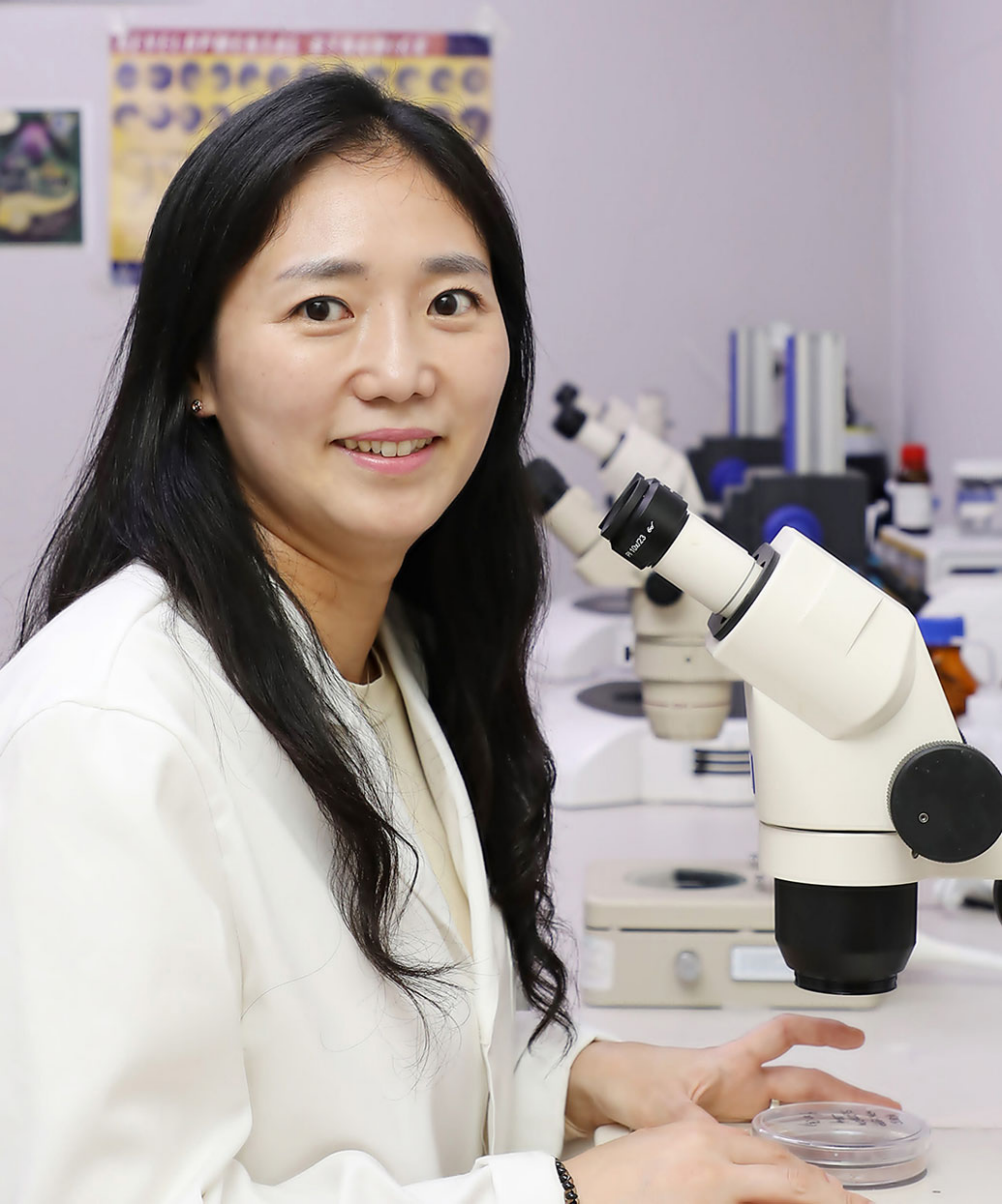


**So-Hyun Lee**

**Who or what inspired you to become a scientist?**

When I was a child, my grandmother became ill and eventually passed away without ever receiving a proper diagnosis. At the time, treatment options were very limited, and diagnostic tools were far less advanced than they are today. The experience of being unable to help her during that difficult time stayed with me and became the driving force behind my decision to pursue science that could improve the lives of patients. Driven by that experience, I chose the path of becoming a scientist and developed a particular interest in the field of research. Eventually, I discovered that zebrafish are a perfect model organism since they are small, transparent and allow real-time observation of brain and neural development. Their unique characteristics make them a powerful tool for studying human neurological diseases and for pursuing discoveries that may one day lead to better treatments.

**What is the main question or challenge in disease biology you are addressing in this paper? How did you go about investigating your question or challenge?**

In this study, we aimed to uncover the functional role of the *slc25a22a* gene in epilepsy, a severe and often treatment-resistant neurological disorder. Early-onset epileptic encephalopathy severely impairs cognitive development and quality of life. Although variants in the human *SLC25A22* gene have been linked to epilepsy, the underlying mechanisms remain unclear. To investigate this, we generated a zebrafish model with a loss-of-function mutation in *slc25a22a* using CRISPR-Cas9. The mutant larvae showed spontaneous seizure-like behaviors, abnormal local field potential activity and elevated intracellular Ca^2+^ propagation, all of which are hallmarks of epilepsy. We then treated the mutants with valproic acid (VPA), a widely used anti-epileptic drug. VPA significantly reduced seizure frequency and severity, confirming the epileptic nature of the observed phenotype. This model offers a powerful platform for studying monogenic epilepsy and testing therapeutic compounds. Our work demonstrates the value of zebrafish in modeling neurological disease in a whole-organism context.

[…] our zebrafish model can help us to understand epilepsy and find better treatments in the future.

**How would you explain the main findings of your paper to non-scientific family and friends?**

Our brain works like a complex electrical system, using tiny signals to help us think, move and feel. Sometimes, the brain sends out too many signals at once, like an overloaded circuit, which can cause seizures. During a seizure, a person might shake, lose awareness or act in unusual ways. When seizures happen repeatedly, it's called epilepsy. Epilepsy can be especially hard for children born with it. In our study, we used zebrafish – small transparent fish that grow quickly and let us observe brain activity in real time. We focused on a gene linked to epilepsy in humans and introduced the same mutation into zebrafish. The fish began showing sudden, abnormal movements, similar to seizures. After treating them with a common epilepsy drug, the movements were reduced. This shows that our zebrafish model can help us to understand epilepsy and find better treatments in the future.

**What are the potential implications of these results for disease biology and the possible impact on patients?**

Our findings have several important implications for both disease biology and patient care. First, by identifying *slc25a22a* as a gene that can cause seizure-like behavior when mutated, we contribute to a better understanding of the genetic underpinnings of epilepsy, particularly in early-onset cases. This expands the catalog of candidate genes involved in epileptic disorders and provides a foundation for more accurate genetic diagnosis. Second, the zebrafish model we developed enables rapid and cost-effective *in vivo* testing of gene function and drug responses. Since the mutant fish exhibited clear seizure-like activity that responded to anti-epileptic medication, this model could be used for high-throughput screening of new therapeutic compounds. This is particularly significant for patients with rare or drug-resistant forms of epilepsy, where tailored treatments are urgently needed. Finally, our study demonstrates how small-animal models can bridge the gap between clinical genetics and therapeutic development. By mimicking a human genetic disorder in zebrafish and observing both symptoms and treatment effects, we are able to translate genetic findings into actionable biological insights. This translational approach holds promise for developing personalized treatment strategies in the future.

**Figure:**
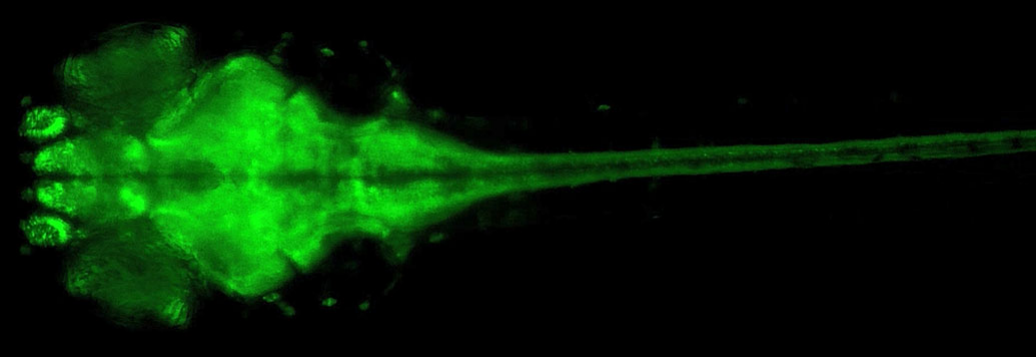
**Increased Ca^2+^ dynamics from the midbrain to the spinal cord in a *slc25a22a* mutant zebrafish.** The elevated fluorescence from GCaMP6s indicates widespread hyperexcitability during spontaneous seizure-like events.

**Why did you choose DMM for your paper?**

We chose Disease Models & Mechanisms (DMM) because it is one of the leading journals that specifically emphasizes the use of model organisms to uncover mechanisms of human disease. Our study, which leverages a zebrafish model to investigate gene function and seizure suppression, fits squarely within this focus. One key factor was that DMM is an open-access journal, which ensures that our research will be freely accessible to the global scientific and medical communities. We felt this would maximize the visibility and impact of our work, encouraging collaboration and accelerating follow-up research. In addition, DMM is known for its balanced editorial approach, valuing both biological rigor and clinical relevance. This made it an ideal venue for presenting our findings as more than a genetic model, but as a contribution to real-world understanding and treatment of epilepsy.

**Given your current role, what challenges do you face and what changes could improve the professional lives of other scientists in this role?**

One of the biggest challenges I face in my current role is the short-term nature of research positions. Many early-career scientists work under temporary contracts tied to specific grants, which creates a sense of job insecurity and makes it difficult to fully commit to long-term, meaningful research. This uncertainty can be especially stressful when trying to plan the next step in one's career or manage personal responsibilities. To improve this situation, it would be beneficial to provide more stable and longer-term research opportunities, particularly for postdoctoral researchers. In addition, encouraging interdisciplinary research environments and promoting international collaboration could help young scientists broaden their perspectives, learn new techniques and build strong professional networks. These opportunities not only support scientific growth but also help researchers develop their careers beyond the lab.

**What's next for you?**

My goal is to improve and expand our zebrafish-based system so that it can become a practical and efficient tool for testing new drugs targeting neurological diseases. I aim to develop an epilepsy drug development system – using our zebrafish model – that incorporates behavior study, electrophysiology and Ca^2+^ imaging. This system would make it possible to quickly identify and test potential new epilepsy drugs. By combining behavior observation, brain activity monitoring and molecular analysis, I hope to identify promising compounds and then test their effects and safety in mice or human organoid models. To support this work, I'm actively seeking international collaborations. Working with researchers from different fields will help me connect what we learn in the lab to real-world treatments. In the future, I hope to contribute to patient-focused therapies by combining model organism research, pharmacology and human data.

**Tell us something interesting about yourself that wouldn't be on your CV**

I try to create a positive and comfortable environment for the people I work with. When research becomes difficult or stressful, I believe that a few kind words or a bit of light humor can really improve the mood. These small, warm moments help encourage good teamwork and keep everyone motivated. I also find balance through exercise. Running and swimming are not just hobbies for me. They help me clear my mind, reset and return to my research with fresh focus and energy.

When people communicate openly, respect each other and work toward the same goals, it not only improves the results but also builds a stronger and more motivated team.

**What keeps you motivated during long or challenging phases of research?**

What helps me keep going during hard times in research is the feeling of connection and teamwork with others. Research can be unpredictable and sometimes disappointing. But being in a place where people support each other by talking about problems, sharing ideas or giving simple encouragement helps me stay steady and focused. I believe that good research often comes from working well as a team. When people communicate openly, respect each other and work toward the same goals, it not only improves the results but also builds a stronger and more motivated team. In this kind of environment, failures feel easier to handle, and progress becomes something we can celebrate together. To me, research is not just something I do alone to find answers but something we build together. That feeling of shared purpose is what keeps me motivated, especially when things get tough.

## References

[DMM052504C1] Lee, S.-H., Liang, T., Chandrasekaran, G., Zhang, J., Kim, S. S., Parvathi, S. V., Lee, S. W., Cho, E.-S., Shin, H.-Y., Yoon, Y.-G. et al. (2025). Mutant zebrafish lacking *slc25a22a* show spontaneous seizures and respond to the anti-seizure medication valproic acid. *Dis. Model. Mech.* 18, dmm052275. 10.1242/dmm.052275 40539845 PMC12208195

